# Comparative analysis of human induced pluripotent stem cell‐derived mesenchymal stem cells and umbilical cord mesenchymal stem cells

**DOI:** 10.1111/jcmm.16851

**Published:** 2021-08-13

**Authors:** Sheeja Rajasingh, Vinoth Sigamani, Vijay Selvam, Narasimman Gurusamy, Shivaani Kirankumar, Jayavardini Vasanthan, Johnson Rajasingh

**Affiliations:** ^1^ Department of Bioscience Research University of Tennessee Health Science Center Memphis Tennessee USA; ^2^ Department of Genetic Engineering SRM Institute of Science and Technology Chennai India; ^3^ Department of Medicine‐Cardiology University of Tennessee Health Science Center Memphis Tennessee USA; ^4^ Department of Microbiology, Immunology and Biochemistry University of Tennessee Health Science Center Memphis Tennessee USA

## Abstract

Generation of induced pluripotent stem cells (iPSCs) and their differentiation into mesenchymal stem/stromal cells (iMSCs) have created exciting source of cells for autologous therapy. In this study, we have compared the therapeutic potential of iMSCs generated from urinary epithelial (UE) cells with the available umbilical cord MSCs (UC‐MSCs). For this, adult UE cells were treated with the mRNA of pluripotent genes (OCT4, NANOG, SOX2, KLF4, MYC and LIN28) and a cocktail of miRNAs under specific culture conditions for generating iPSCs. Our non‐viral and mRNA‐based treatment regimen demonstrated a high reprogramming efficiency to about 30% at passage 0. These UE‐iPSCs were successfully differentiated further into ectoderm, endoderm and mesoderm lineage of cells. Moreover, these UE‐iPSCs were subsequently differentiated into iMSCs and were compared with the UC‐MSCs. These iMSCs were capable of differentiating into osteocytes, chondrocytes and adipocytes. Our qRT‐PCR and Western blot data showed that the CD73, CD90 and CD105 gene transcripts and proteins were highly expressed in iMSCs and UC‐MSCs but not in other cells. The comparative qRT‐PCR data showed that the iMSCs maintained their MSC characteristics without any chromosomal abnormalities even at later passages (P15), during which the UC‐MSCs started losing their MSC characteristics. Importantly, the wound‐healing property demonstrated through migration assay was superior in iMSCs when compared to the UC‐MSCs. In this study, we have demonstrated an excellent non‐invasive and pain‐free method of obtaining iMSCs for regenerative therapy. These homogeneous autologous highly proliferative iMSCs may provide an alternative source of cells to UC‐MSCs for treating various diseases.

## 1 INTRODUCTION

Mesenchymal stem/stromal cells (MSCs) are multipotent adult stem cells possess a limited proliferation potential but extensive differentiation and self‐renewal capacities in vitro and in vivo. MSC capabilities of multipotency, expandability, hypo‐immunogenicity and the immunoregulatory properties are promising for tissue regeneration.^1^ Studies have shown that MSCs have a great ability to undergo trilineage differentiation into osteocytes, chondrocytes and adipocytes.^2^, ^3^ MSCs can be obtained from many different tissues such as cord blood, bone marrow, adipose tissue or other connective tissues.^4^ Currently, MSCs have been identified as a valuable cell source for therapy including the characteristics of immunomodulation, angiogenesis, anti‐apoptosis, anti‐fibrotic and chemo‐attractive activities.^5^, ^6^ Moreover, the paracrine factors secreted from MSCs are facilitated to support the growth and differentiation of neighbouring cells to where it is transplanted. However, adult MSCs derived from majority of the sources have a limited proliferative capacity and with a heterogeneous cell population.

Among stem cells, MSCs are considered to have a wide range of therapeutical applications. MSCs have some unique biological abilities because of their immunomodulatory and regenerative therapeutic potentials.^7^ MSCs also have the ability to modulate the humoral and cellular responses.^8^ Furthermore, MSCs have the potential to secrete anti‐inflammatory cytokines and chemokines which makes them suitable for treating autoimmune disorders.^9^, ^10^, ^11^ Importantly, MSCs do not have class II antigen expression which is good for allogenic cell transplantation.^12^ As interest grows, there are some critical issues with the use of MSCs for clinical treatment. First, MSCs were identified in bone marrow and later from several other sources.^4^ Though adult MSCs can be obtained from various tissues, the number of cells available for therapy is still a major challenge, and the procedures for collecting the cells are highly invasive and painful.

In many countries, at during deliveries, the parents are advised to preserve the umbilical cord or blood in stem cell banks for their offspring for the future purpose of autologous stem cell therapy, if the need arises. Most of these stem cell banks offer storage facilities only for a limited time, usually for about 15–25 years and are very expensive. Moreover, several diseases are being revealed only after the age of 40, and the stem cells stored in the bank may not be suitable for use, especially after a prolonged storage. The discovery of MSCs derived from the controlled differentiation of induced pluripotent stem cells (iPSCs) would be an alternative source for obtaining a homogeneous population of MSCs for therapy.^13^ In this study, we have generated iPSC‐derived MSCs from urinary epithelial cells (referred as iMSCs) which are isolated from human urine samples. Our novel non‐invasive approach of generating iMSCs will be a good source of autologous cells for regenerative disease therapy. With this simple promising non‐invasive method, we have generated a high‐quality, autologous iMSCs with a high replicative potential which are suitable for the regenerative therapy. Moreover, we compare the therapeutic efficiency of the generated human iMSCs with umbilical cord MSCs (referred as UC‐MSCs).

## 2 MATERIALS AND METHODS

### 2.1 Antibodies and reagents

We used primary antibodies for OCT4, NANOG, SOX2 (Cell Signaling Technology), β‐actin, TRA1‐60, SSEA4, VE‐Cadherin, α‐fetoprotein (AFP) (Santa Cruz Biotechnology, Inc.) and Nestin (R & D Systems), to perform in vitro analysis. More details about the used antibodies are given in Table S1. Secondary antibodies APC‐. TRITC‐, PE‐ and FITC‐conjugated donkey anti‐mouse, anti‐mouse, anti‐goat and anti‐rabbit (Jackson ImmunoResearch Laboratories, Inc.) were used. We used NutriStem (NS) medium, reprogramming kits, alkaline phosphatase assay kits (Repromed) and DAPI (Life Technologies).

### 2.2 Institutional regulatory approval

This study protocol is approved by the UTHSC Institutional Review Board (IRB). All methods pertaining to human samples were carried out in accordance with the relevant guidelines, and regulations were approved by the IRB of UTHSC (19‐07027‐XP dated 01/14/2020), Memphis, TN and the IRB from my previous affiliated institution, the University Kansas Medical Center, Kansas City, KS (141900 dated 09/13, 2018).

### 2.3 Urinary epithelial (UE) cell culture

We have collected urine sample after obtained written informed consent from a 55‐year old male healthy volunteer. To isolate and culture the urinary epithelial (UE) cells from urine, we have employed a modified the protocol as described by others earlier.^14^ Briefly, UE cells were isolated by centrifuging 150–200 ml of urine at 500 *g* for 10 min at room temperature. The pellet containing the UE cells was cultured in a 25‐ml flask containing DMEM complete medium containing 15% FBS medium at 37℃ in a 5% CO_2_ incubator with humidified air. The isolated UE cells were identified by the protein expression of CK19 and ZO1. When these cells become 80% confluent, they were sub‐cultured and used for reprogramming experiments.

The human UC‐MSCs were purchased from Sciencell Research Laboratories. The neonatal human foreskin fibroblasts (NUFF) from Stemgent, and human umbilical vein endothelial cells (HUVECs) were purchased from American Type Culture Collection (ATCC). These cells were cultured and maintained as per supplier's instruction.

### 2.4 Non‐viral reprogramming of human UE cell‐derived iPSCs

When the UE cells attained 80% confluent, they were sub‐cultured into a 6‐well plate coated with iMatrix (Reprocell USA Inc) with NS medium. Then, the UE cells were reprogrammed with the mRNA of OCT4, NANOG, SOX2, KLF4, MYC and LIN28 by using transfection agent, Lipofectamine along with a cocktail of microRNAs (Reprocell USA Inc) for 10 days as described in our earlier publications^15^, ^16^ From day 9, we have observed several iPSC‐granulated colonies resembled human embryonic stem cell colonies. These iPSC colonies were identified by TRA1‐60 live staining, and the positive colonies were manually picked and further cultured on Matrigel‐coated plates in NS medium at 37℃ in a 5% CO_2_ incubator with humidified air. These cells were used for further experiments.

### 2.5 Alkaline phosphatase staining

The iPSCs were cultured in a 4‐well dish for three days. The cells were washed twice with phosphate‐buffered saline (PBS). Then, the cells were fixed with the fix solution for 2–5 min and again washed twice with PBS. Staining solution from the alkaline phosphatase kit (Stemgent, # 00‐0055) was added to each well and incubated in the dark at room temperature for 5–15 min. The reaction was stopped by aspirating the solution and washing the wells twice with 2 mL of PBS. Stained colonies were observed under the microscope.

### 2.6 Differentiation of iPSCs into endoderm cells

For the endoderm differentiation, UE‐iPSCs were cultured in NS medium in a 30‐mm culture dish. When the cells reached 70%–80% confluent, the NS medium was removed and replaced with the Stemdiff definitive endoderm medium (Stemcell Technologies) and cultured again for 14 days. We have observed that the culture displayed significant morphological changes including the cuboid cell shape of primary hepatocytes. These day 14 cells were used for qRT‐PCR analysis for the mRNA expression of hepatocyte markers apolipoprotein A1 (APOA1) and α−fetoprotein (AFP) and immunofluorescence analysis for AFP protein expression.

### 2.7 Differentiation of iPSCs into neuronal cells

For the differentiation of human UE‐iPSCs into neuronal cells, the UE‐iPSCs were cultured in NS medium in a 30‐mm culture dish. When the cells become 70%–80% confluent, the NS medium was removed and replaced with the neuronal induction medium and cultured again for 18 days (Stemcell Technologies). We have observed the changes in cell morphology and displayed the neuronal‐like cells from day 14 onwards. The cells were collected on day 18 for the mRNA expression of neuronal‐specific genes OLIG2 and MAP2 by qRT‐PCR analysis and protein expression of Nestin by immunofluorescence analysis.

### 2.8 Differentiation of iPSCs into mesoderm cells

For the differentiation of human iPSCs into mesoderm cells specifically endothelial cells (ECs), we used the protocol as described by us earlier.^15^ Briefly, the iPSCs were plated in a 30‐mm culture dish in NS medium. When the cells obtain 80% confluence, the cells were cultured in mesodermal medium (DMEM supplemented with 1X B27, 1X N2, 5 μM CHIR, 25 ng BMP4) for 3 days. Then, the cells were cultured in the StemPro34 medium for 4 days. The cells were allowed to grow in endothelial EGM2 medium until they became mature ECs. These ECs were further confirmed by the mRNA and protein analyses.

### 2.9 Differentiation of human UE‐iPSCs into iMSCs

For the differentiation of UE‐iPSCs into iMSCs, we cultured and maintained UE‐iPSCs in NS medium. When the cells reached 70%–80% confluency, the NS medium was removed and fresh mesenchymal induction medium (STEMdiff‐ACF, Stem Cell Technologies) was added to the plates for 4 days followed by MesenCult ACF Plus medium for 21 days, during which the medium was changed once in every two days. These cells were further maintained and sub‐cultured in MesenCult ACF plus medium in a 5% CO_2_ incubator at 37℃. When the cells reached 80% confluent, they were sub‐cultured using Typsin LE for further experiments.

### 2.10 Trilineage differentiation of iMSCs

#### 2.10.1 Differentiation of iMSCs into osteocytes

The iMSCs were seeded into a 30‐mm culture dish at a density of 7.5 × 10^5^ cells with the MesenCult ACF Plus medium. After 24 h, the MesenCult medium was removed, and 2 ml of osteocyte differentiation (OD) medium which contains alpha MEM medium supplemented with ascorbic acid (50 µg/ml), β‐glycerophosphate (5 mM), 20% of FBS, 1% GlutaMAX and 1% of penicillin/streptomycin was added. After 7 days, the OD medium was replaced with osteocyte mineralization medium (OD medium with 10 nM dexamethasone) for another 14 days. The medium was changed once in every two days. After 21 days, the induced osteocytes (iOST) were collected and used for further mRNA and protein analyses.

#### 2.10.2 Differentiation of iMSCs into chondrocytes

The iMSCs were cultured in a 30‐mm culture dish at a density of 7.5 × 10^5^ cells in MesenCult ACF Plus medium. After 24 hours, the MesenCult medium was removed, and 2 ml of chondrocyte differentiation medium (Thermo Fisher) was added for 17 days. The medium was changed once in every two days. These induced chondrocytes (iCHON) were collected after day 17 and were characterized by mRNA and protein analyses.

#### 2.10.3 Differentiation of iMSCs into adipocytes

For the adipocyte differentiation, 7.5 × 10^5^ iMSCs were seeded into a 30‐mm culture dish with MesenCult ACF Plus medium. After 24 h, the MesenCult medium was removed, and 2 ml of adipocyte differentiation medium (Thermo Fisher) was added to the cells and was cultured for 11 days with the regular media change at every two days. These induced adipocytes (iADIPO) were collected after day 11 and were further characterized by mRNA and protein analyses.

### 2.11 Quantitative RT‐PCR analysis

To characterize the iPSCs and iMSCs, we have performed quantitative RT‐PCR (qPCR) for studying the gene expression pattern as described in our earlier publications.^15^ Briefly, the RNA was collected from the cells by adding TRIzol reagent (Ambion by Life Technologies). The list of SYBR green primers and TaqMan primers used in this study is available in Tables S2 and S3. The quantification of these RNA samples was performed in the NanoDrop 8000 Spectrophotometer (Thermo Fisher). The relative mRNA expression of the targeted genes was normalized to the 18S rRNA as endogenous control. Results were expressed as fold change, and the values were calculated as the ratio of induced expression to control expression.

### 2.12 Western blot analysis

The Western blot analysis for the protein expression was performed in the UE‐iPSCs and iMSCs as described in our earlier publications.^17^, ^18^ Briefly, the cells were collected and centrifuged for protein isolation. After centrifugation, 50 μl of lysis buffer was added to the cell pellet. Then, the samples were centrifuged at 12,000 *g* for 20 min at 4℃. The supernatant was carefully removed without disturbing the pellet. The isolated protein samples were quantified by Bradford's method using the AccurisTM instrument SmartReader 96‐well microplate absorbance reader at 595 nm. Equal amount of proteins was calculated and loaded into a SDS‐PAGE. After the electrophoresis is completed, the proteins from the gel were transferred into a PVDF membrane. Immunoblotting was performed using a specific primary and secondary antibody, followed by visualization of protein bands using LI‐COR Phosphorimager (Odyssey) and analysed using the Image Studio Lite software.

### 2.13 Flow cytometry analysis

Flow cytometry analysis was performed to characterize the UE‐iPSCs and iMSCs phenotypes as described by us earlier.^19^ Briefly, cells from a six‐well plate were harvested and washed twice in phosphate‐buffered saline (PBS). The cells (1 × 10^6^ cells/sample) were initially incubated with 5% donkey serum for 30 min at 4℃. Subsequently, cells were incubated with the appropriate primary antibodies for 1 h at room temperature in a rocker. Then, the cells were washed with PBS three times and then incubated for 20 min with an appropriate secondary antibody at 37℃. Appropriate isotype controls were used for all the experiments. Finally, the cells were washed three times with PBS and resuspended in 0.5 ml of PBS. The cells were analysed by flow cytometer (FACSCalibur, BD Biosciences) using Cell Quest software. Data were analysed by using FlowJo software (Tree Star).

### 2.14 Immunofluorescence staining

The immunofluorescence staining was performed to analyse the protein expression as described by us earlier.^15^, ^18^ Briefly, the cells reached 80% confluency, and they were washed with PBS and fixed using 4% paraformaldehyde for 3 min. The cells were rinsed with PBS for three times at one‐minute internals of each wash. Blocking was done using 5% donkey serum and then incubated with the primary antibodies for overnight at 4℃. On the following day, fluorescence‐tagged secondary antibodies specific to the host and primary antibodies were added and incubated at 37℃ for 1 h. Nuclear staining was done by using DAPI (Molecular Probes, Life Technology), and the cells were covered by using a glass coverslip. The stained slides were visualized using an inverted fluorescence microscope (OlympusIX71), and the images were captured using CellSens standard software.

### 2.15 Scratch assay

First, the migration potentials of iMSCs and UC‐MSCs were evaluated using scratch assay as described earlier.^20^ Secondly, the migration capacity of condition medium from iMSCs and UC‐MSCs was analysed by using human neonatal foreskin fibroblasts (NUFF) and human umbilical vein endothelial cells (HUVECs). These iMSCs, UC‐MSCs, NUFF and HUVEC were cultured in their appropriate media. Once the cells reached 70%−80% confluency, they were harvested, and a suspension of 20,000 cells was seeded on the culture inserts placed in 30‐mm dishes. After 24 h, the inserts were removed, and this type of insert forms a homogeneous cell‐free lane in the middle of the confluent monolayer of cells. Immediately after the removal of inserts, the resulting scratches were pictured under phase‐contrast microscope and live imaging microscope (T0). After imaging, the cells were further cultured for another 24 h in the presence or absence of conditioned medium from UC‐MSCs or iMSCs (T24). The cell migration between the scratch was video recorded using live imaging or imaged under phase‐contrast microscope. The rate of cell migration was calculated using ImageJ software (NIH) by measuring the cell area covered in T0 and T24. The results were expressed in percentage of wound closure under specific conditioned medium.

### 2.16 Transwell migration assay

Migration capacity of the UC‐MSCs and iMSCs was evaluated using 24‐well Transwell insert containing transparent polyester membrane having 8‐micron pore size (Corning). A total of 75 × 10^3^ cells per well were seeded in inserts of each Transwell plate in FBS‐free media. The wells hold the inserts, and 0.5 ml of DMEM supplemented with 10% FBS as attractant was added. The plates were incubated at 37℃ in a 5% CO_2_ incubator for 48 h. The cells that migrated to the surface of the wells were stained with 0.5% crystal violet in 2% methanol for 20 min. The attached cells in the wells were washed in PBS for three times to remove excess dye. The number of stained cells was counted using an inverted phase‐contrast microscope. The degree of migration was expressed as the number of migrated cells per 10× microscopic visual field (mvf).

### 2.17 Karyotyping analysis

To determine the cell integrity and chromosomal abnormalities, G‐banded karyotyping analysis was performed using 15^th^ passage iMSCs and UC‐MSCs at the WiCell Inc, Madison, WI.

### 2.18 The colony‐forming unit (CFU) assay

The iMSCs and UC‐MSCs were seeded into 30‐mm culture dishes at a density of 6.25 × 10^4^ cells in MesenCult medium (MesenCult™ Proliferation Kit, Stemcell Technologies) for 14 days. After 7 days of culture, small‐ to medium‐sized colonies were visible and observed. The day 14 cultures were stained by crystal violet to distinguish colonies. For the crystal violet staining, the cells were placed on ice and washed with cold PBS and then fixed for 10 min with ice‐cold 100% methanol. Then, the cells were incubated with 0.05% crystal violet solution in 25% methanol for 20 min. Finally, the cells were washed with water for 5 times, the colonies were counted and the images were taken by the EVOS phase‐contrast microscope.

### 2.19 Telomere length quantification

To analyse the absolute telomere length in different iMSCs, the cells were collected in different passages, and its DNA was isolated by DNeasy kit (Qiagen). The isolated DNA was analysed using Absolute Human Telomere Length Quantification qPCR Assay Kit (ScienCell Research Laboratories).

### 2.20 Statistical analysis

All experiments were repeated at least three times. Results are presented as mean ± SD. Comparisons were performed by one‐way ANOVA (GraphPad Prism), and probability values less than 0.05 were considered as statistically significant.

## 3 RESULTS

### 3.1 Non‐viral and safe method of reprogramming human UE cells into iPSCs

**FIGURE 1:**
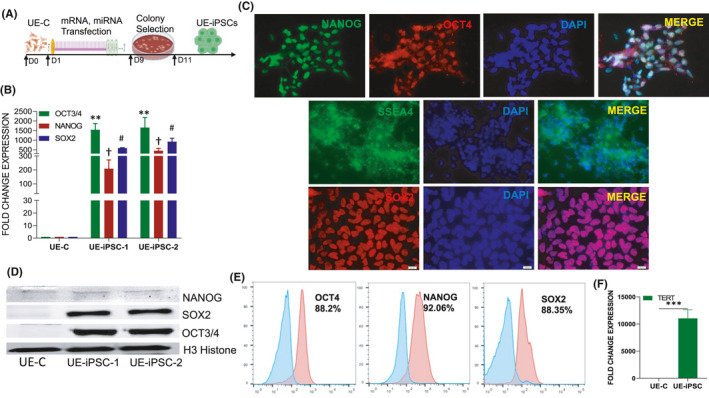
Characterization of the pluripotency of UE‐iPSCs. (A) Schematic illustration of the generation of urinary epithelial (UE)‐derived iPSCs through transfection with cocktail of mRNA and miRNA on day 1 through day 9, colony selection on day 9 and identification of UE‐iPSC by day 11. (B) mRNA expression of pluripotent genes OCT4, NANOG and SOX2 was examined by qRT‐PCR in two clones of UE‐iPSCs and UE control (UE‐C) cells served as control. The mRNA expression was normalized with 18S rRNA. **p < 0.001 OCT4 vs. UE‐C; ^†^ *p *< 0.001 NANOG vs. UE‐C; ^#^ *p *< 0.001 SOX2 vs. UE‐C. (C) Protein expression was analysed by immunofluorescence staining of OCT4, NANOG, SOX2 and SSEA4. DAPI staining was done to visualize the nucleus. (D) Western blotting analysis for the expression of OCT4, NANOG and SOX2 in two different clones of UE‐iPSCs (UE‐iPSC‐1 and UE‐iPSC‐2). H3 histone in Western blotting was used as loading control. (E) Flow cytometric analyses for the expression of pluripotency proteins NANOG, OCT4 and SOX2 in UE‐iPSCs. The cells stained with secondary antibody alone were used as negative control. (F) The mRNA expression of telomerase reverse transcriptase (TERT), the enzyme which increases the length of telomeres, was studied in UE‐iPSCs and UE‐C, which were used as controls. The mRNA expression of TERT gene is significantly increased in UE‐iPSCs when compared to UE‐C. ***p < 0.0001 vs. UE‐C

Human UE cells were isolated from the urine sample and characterized by the expression of proteins CK19 and ZO1 (Figure S1A). These UE cells were cultured and maintained in a 25‐ml cell culture flask containing complete DMEM. When these cells became 80% confluent, the cells were sub‐cultured in a 6‐well plate coated with iMatrix in NutriStem medium. At 70% cell confluency, the cells were transfected with pluripotent genes using a StemRNA Reprogramming kit for 10 days as described in our earlier publications.^15^, ^16^ The protocol we have used for reprogramming UE cells into iPSCs is represented in the schematic illustration (Figure 1A). Phase‐contrast microscopic images of sequential changes were observed on day 0, day 5, day 7 and day 9 during reprogramming of UE cells into iPSCs (Figure S1B). From day 9, we have observed that several iPSC‐granulated colonies and cells containing large‐size nuclei that were occupied the maximum area in the cytoplasm. The iPSC‐positive colonies were identified by TRA1‐60 live staining. The positive colonies were manually picked under the phase‐contrast microscope and grown in new culture dishes (Figure S1C). Furthermore, these iPSCs were confirmed by alkaline phosphatase staining (Figure S1D). To quantify the reprogramming efficiency, we performed flow cytometry analysis and our data showed that 29.1% of cells were reprogrammed into iPSCs by expressing the important pluripotent proteins OCT4 and SOX2 (Figure S1E) at passage 0 (P0). These UE‐iPSCs that were above P7 were used for further experiments.

### 3.2 Characterization of UE‐iPSCs

Two colonies of UE‐iPSCs were characterized; the qRT‐PCR analysis data showed a significantly increased level of mRNA expression of pluripotent genes OCT4, NANOG and SOX2 when compared to non‐reprogrammed UE control (UE‐C) cells (Figure 1B). The enhanced mRNA‐specific pluripotent gene expressions were further corroborated by immunofluorescence staining of pluripotent proteins OCT4, SOX2 and SSEA4 (Figure 1C). We also characterized the UE‐iPSCs by Western blot analysis and confirmed that the reprogrammed UE‐iPSCs were showing prominent protein bands for OCT4, SOX2 and NANOG (Figure 1D). Our flow cytometric analyses further quantified that the UE‐iPSCs showed more than 88% cells were expressing pluripotency proteins OCT4, NANOG and SOX2 (Figure 1E). The mRNA expression of telomerase reverse transcriptase (TERT), an enzyme which increases the length of telomeres, was found significantly increased in UE‐iPSCs (Figure 1F). This increased TERT expression demonstrated that the generated UE‐iPSCs were having the potential of proliferation. Overall, our results strongly demonstrated that the iPSCs generated from the UE cells were truly proliferative human pluripotent stem cells. With this approach, we have generated six bona fide iPSC colonies and two iPSC colonies were used for further experiments.

### 3.3 Confirmation of pluripotency by in vitro differentiation analysis

**FIGURE 2:**
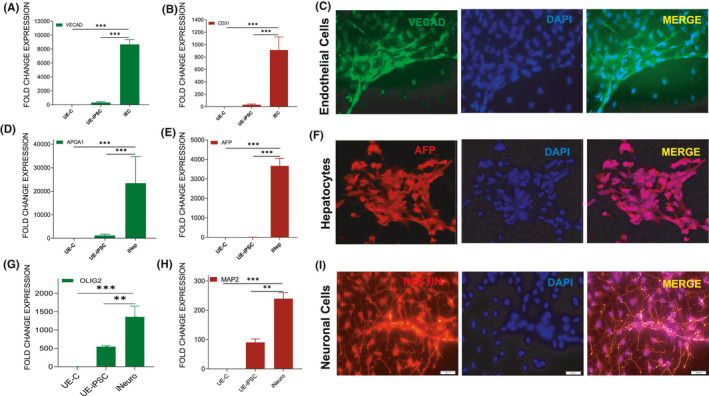
In vitro trilineage differentiation of pluripotent UE‐iPSC analysis. (A–C) UE‐iPSCs were cultured in a mesoderm‐specific medium for 21 days followed by qRT‐PCR analysis for the expression of mesodermal (endothelial cells) genes (A) VE‐cadherin and (B) CD31 in the differentiated culture when compared to the UE‐iPSCs and UE‐parent cells (UE‐C). ***p < 0.0001 vs. UE‐C or UE‐iPSCs. (C) Immunofluorescence analysis for the protein expression of VE‐cadherin (green) in the mesodermal differentiated cultures. DAPI (blue) was used to visualize the nucleus. (D–F) UE‐iPSCs were cultured in Stemdiff definitive endoderm medium for 14 days followed by qRT‐PCR analysis for the expression of endodermal (hepatocytes) genes. (D) Apolipoprotein A1 (APOA1) and (E) α−fetoprotein (AFP) in the differentiated culture when compared to the UE‐iPSCs or UE‐parent cells (UE‐C). (F) Immunofluorescence analysis for the AFP (red) protein expression in the differentiated culture. DAPI (blue) was used to visualize the nucleus. ***p < 0.0001 vs. UE‐C or UE‐iPSCs. (G–I) UE‐iPSCs were cultured in neuronal‐specific induction medium for 18 days followed by qRT‐PCR analysis for the expression of neural (G) OLIG2 and (H) MAP2 genes in the differentiated culture when compared to the UE‐iPSCs and UE‐C. ***p < 0.0001 vs. UE‐C and **p < 0.001 vs. UE‐iPSC. (I) Immunofluorescence analysis for the Nestin (red) protein expression in the differentiated culture (I). DAPI (blue) was used to visualize the nucleus

We have further studied the in vitro differentiation potentials of UE‐iPSCs into mesoderm, endoderm and ectoderm lineage cells to prove the efficiency of their pluripotency. For the mesodermal lineage differentiation, we have cultured the UE‐iPSCs in a mesoderm‐specific culture medium for 21 days as described by us earlier.^15^ Our qRT‐PCR analyses demonstrated a significantly increased expression of endothelial cell (EC) genes CD31 and VE‐cadherin in the mesodermal differentiated culture compared with the UE‐iPSCs and UE‐C cells (Figure 2A,B). Further, immunofluorescence analyses confirmed the expression of VE‐cadherin protein in the EC differentiated culture (Figure 2C). For the endodermal lineage differentiation, we have cultured the iPSCs in Stemdiff definitive endoderm‐specific medium. We have observed that the endoderm differentiation culture displayed significant morphological changes such as primary hepatocytes. The day 14 cells were collected, and qRT‐PCR analysis was performed for the hepatocyte‐specific markers apolipoprotein A1 (APOA1) and α‐fetoprotein (AFP). Our qRT‐PCR analysis demonstrated a significantly increased expression of hepatocyte genes APOA1 and AFP in the differentiated culture when compared to the UE‐iPSCs and UE‐C cells (Figure 2D,E). The immunofluorescence analysis further confirmed the expression of AFP in the hepatocyte differentiation culture (Figure 2F). For differentiation towards ectodermal lineage, we have cultured the UE‐iPSCs with neuronal induction medium. These cells were collected on day 18, and qRT‐PCR analysis was performed for the expression of neuronal‐specific markers OLIG2 and MAP2. Our qRT‐PCR analysis data demonstrated a significantly increased expression of neuronal‐specific genes OLIG2 and MAP2 in the neuronal differentiation culture when compared to the UE‐iPSCs and UE‐C cells (Figure 2G,H). Moreover, the immunofluorescence image analysis data demonstrated the expression of Nestin in the differentiated cells (Figure 2I). These data clearly demonstrated that the UE‐iPSCs are true pluripotent stem cells and were capable of being differentiated into any kind of cells in the human body.

### 3.4 Generation and characterization of iMSCs derived from UE‐iPSCs

**FIGURE 3:**
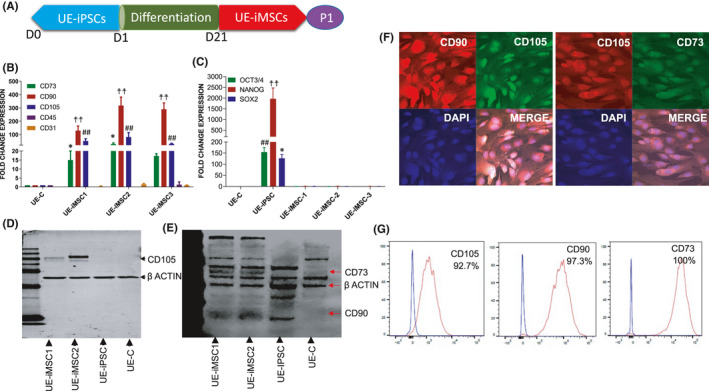
Generation and characterization of UE‐iPSC‐derived MSCs (iMSC). (A) Schematic illustration of the generation of iMSCs derived from UE‐iPSCs. UE‐iPSCs were cultured in NutriStem XF/FF culture medium followed by mesenchymal induction medium (STEMdiff‐ACF Mesenchymal Induction Medium) for 21 days to obtain iMSCs. (B) qRT‐PCR for the expression of mesenchymal specific genes CD73, CD90, CD105 and no or low expression of hematopoietic markers CD34 and CD45 in three different clones of iMSCs (iMSC‐1, iMSC‐2 and iMSC‐3) when compared to the parent cells (UE‐C). The mRNA expression was normalized with 18S rRNA. **p < 0.01 CD73 vs. UE‐C; ^††^ *p *< 0.001 CD90 vs. UE‐C; ^##^ *p *< 0.01 CD105 vs. UE‐C. (C) qRT‐PCR results showed that no expression of iPSC markers OCT4, NANOG and SOX2 in three different clones of iMSCs (iMSC‐1, iMSC‐2 and iMSC‐3) when compared to the UE‐iPSC. The mRNA expression was normalized with 18S rRNA. **p < 0.01, ^††^ *p *< 0.001, ^##^ *p *< 0.01 vs. UE‐C, iMSC‐1, iMSC‐2 and iMSC‐3. (D and E) Western immunoblotting for the expression of proteins CD73, CD90 and CD105 in two different clones of iMSCs (iMSC‐1 and iMSC‐2). The UE‐iPSCs and UE‐C cells did not express these MSC‐specific proteins. Actin was used as a loading control. (F) Immunofluorescence analysis for the expression of mesenchymal markers CD73, CD90 and CD105 in iMSCs. (G) Flow cytometric analysis for the expression of mesenchymal surface markers CD73, CD90 and CD105 in iMSCs. More than 92% cells are expressing either CD73 or CD90 or CD105. Isotype control antibodies were used as negative control

We have examined the differentiation potential of UE‐iPSCs into iMSCs in vitro. For this, UE‐iPSCs were cultured in mesenchymal induction medium for 21 days. The iMSCs were then maintained and sub‐cultured in MesenCult ACF medium (Figure 3A). Phase‐contrast microscopic images displayed the sequential changes occurring from day 0 to day 22 during the differentiation of iPSCs into MSCs (Figure S2A–F). The iMSC morphology at different passages was also observed under microscopic images (Figure S2G–L). To characterize the generated iMSCs, we performed quantitative real‐time PCR (qRT‐PCR) analysis for specific genes CD73, CD90 and CD105 as positive markers and CD34 and CD45 as negative markers as described in our earlier publications.^18^, ^21^ The qRT‐PCR data showed that the expression of CD73, CD90 and CD105 was significantly increased in three different clones of iMSCs (iMSC‐1, iMSC‐2 and iMSC‐3) when compared to the UE‐C cells (Figure 3B). Moreover, these iMSCs were showing low or no expression of the negative markers CD34 and CD45 (Figure 3B), and at the same time, the iPSC markers (OCT3/4, NANOG and SOX2) were absent in all three clones of iMSCs (Figure 3C). Our Western immunoblotting results further confirmed the constitutive expression of CD73, CD90 and CD105 in two different clones of iMSCs (Figure 3D,E). The enhanced mRNA expression of MSC‐specific genes was further corroborated by immunofluorescence staining of high‐level expression of mesenchymal markers CD73, CD90 and CD105 in iMSCs (Figure 3F). Our flow cytometric analysis demonstrated that the triple positivity for the mesenchymal markers CD73, CD90 and CD105 was significantly expressed in higher number of cells in both the iMSC clones (Figure 3G). Overall, our results have demonstrated that the generated iMSCs have satisfied all the requirements to resemble human adult MSCs.

### 3.5 Trilineage differentiation potential of iMSCs

**FIGURE 4:**
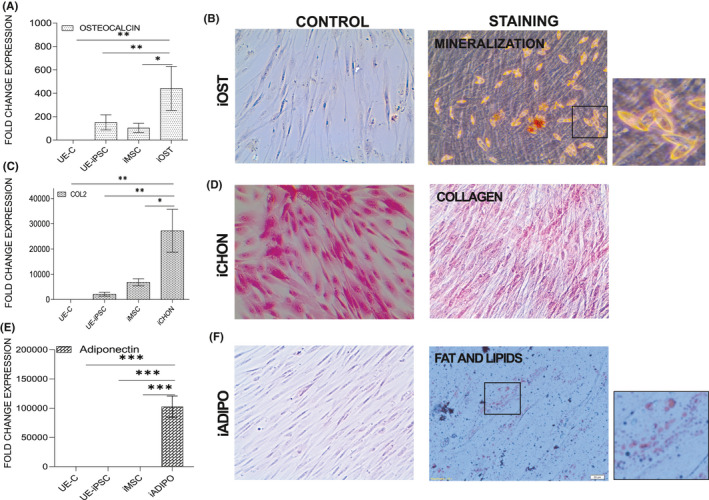
Trilineage differentiation potential of iMSCs into induced osteocytes (iOST), induced adipocytes (iADIPO) and induced chondrocytes (iCHON) lineages. (A) iMSCs were cultured in osteocyte‐specific differentiation medium. After 7 days, the cells were cultured in osteocyte mineralization medium until day 21. The day 21 osteocytes were collected and performed the qRT‐PCR for the expression of osteocyte‐specific gene osteocalcin in the iOST, compared with iMSC or UE‐iPSC or UE‐C. The mRNA expression was normalized with 18S rRNA. *p < 0.01 osteocalcin vs. iMSCs and UE‐iPSCs; **p<0.001 osteocalcin vs. UE‐C. (B) Calcium deposits in the iOST were identified by alizarin red staining. The mineralized regions in iOST were stained positive as red. (C) iMSCs were cultured in the chondrocyte differentiation medium for 17 days. On day 17, qRT‐PCR analyses were performed for the expression of chondrocyte‐specific collagen‐2 gene expression in iCHON when compared with iMSC or UE‐iPSC or UE‐C. *p < 0.01 COL2 vs. iMSCs; **p<0.001 COL2 vs. UE‐C and UE‐iPSCs. (D) Collagen deposition in the iCHON was identified by haematoxylin and eosin staining. Haematoxylin and eosin staining showed the collagen present in the chondrocytes as pale pink in colour, whereas the cell containing cytoplasm stained red or dark pink by the eosin Y, and the haematoxylin stained nucleus as blue. (E) iMSCs were cultured in the adipocyte differentiation medium for 11 days. qRT‐PCR was performed for the expression of adipocyte‐specific adiponectin gene expression in iADIPO when compared with iMSC or iPSC or UE‐C. ***p < 0.0001 adiponectin vs. iMSCs, UE‐iPSCs and UE‐C. (F) The deposition of fat and lipid droplets in the iADIPO was identified by staining with Oil Red O solution

The trilineage differentiation potentials of iMSCs are the unique property and a key requirement for identifying and characterizing the MSC population.^22^, ^23^ Henceforth, we have determined the in vitro trilineage differentiation potentials of iMSCs into osteocyte, adipocyte and chondrocyte lineages in order to prove the generated iMSCs were true population of MSCs. For the differentiation of iMSCs into induced osteocytes (iOST), iMSCs were cultured in the osteocyte differentiation medium. After 7 days, the cells were cultured in osteocyte mineralization medium until day 21. The sequential morphological changes from iMSC to iOST were observed under a phase‐contrast microscope (Figure S3A–C). After 21 days, the osteocytes were collected for further analyses. Our qRT‐PCR results show that osteocyte‐specific osteocalcin gene expression was significantly increased in iOST, compared with iMSC or UE‐iPSC or UE‐C cells (Figure 4A). Furthermore, calcium deposits in the iOST were identified by alizarin red staining (Figure 4B). The mineralized regions in iOST were stained positive as red (Figure 4B). For the differentiation of iMSCs into induced chondrocytes (iCHON), iMSCs were cultured in the chondrocyte differentiation medium for 17 days. The sequential morphological changes from iMSCs to iCHON which were similar to regular chondrocytes were observed under a phase‐contrast microscope (Figure S3D–F). These iCHON were collected for further mRNA and protein analysis. qRT‐PCR results showed that chondrocyte‐specific collagen‐2 gene expression was significantly increased in iCHON, compared with iMSC or UE‐iPSC or UE‐C cells (Figure 4C). Furthermore, collagen deposition in the iCHON was identified by haematoxylin and eosin staining. Haematoxylin and eosin staining showed that the collagen present in the chondrocytes as pale pink in colour and the cytoplasm stained in red or dark pink by the eosin Y, and haematoxylin stained the nucleus as blue (Figure 4D). For the differentiation of iMSCs into induced adipocytes (iADIPO), iMSCs were cultured in adipocyte differentiation medium for 11 days. The sequential morphological changes from iMSCs to iADIPO were observed under a phase‐contrast microscope (Figure S3G–I). These iADIPO were collected for further analyses. Our qRT‐PCR results showed that adipocyte‐specific adiponectin gene expression was significantly increased in iADIPO, compared with iMSC or UE‐iPSC or UE‐C cells (Figure 4E). Furthermore, the deposition of fat and lipid droplets in the iADIPO was identified by staining with Oil Red O solution (Figure 4F). These data clearly showed that the iMSCs generated from UE cells were true‐positive MSCs.

### 3.6 Comparison of iMSCs and UC‐MSCs in relation to the expression of mRNA and proteins

**FIGURE 5:**
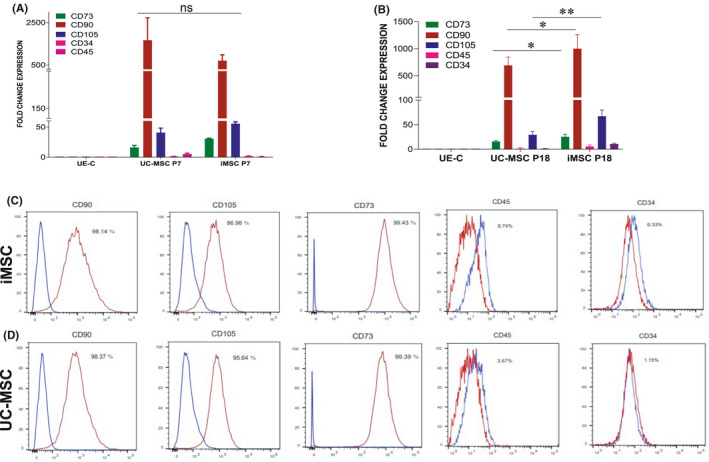
Comparison of mesenchymal cell surface marker expressions in iMSCs and UC‐MSCs. (A) We examined the qRT‐PCR analysis for the expression of mesenchymal specific genes CD73, CD90 and CD105 and mesenchymal negative markers CD34 and CD45 in iMSCs compared with UC‐MSCs in early passage (P7). There are no significant changes observed between iMSCs and UC‐MSCs in the expression of MSC‐specific genes at early passage, ns—not significant. (B) We also performed the qRT‐PCR analysis for the expression of mesenchymal marker genes at a higher passage (P18) in iMSCs and UC‐MSCs. (C and D) Flow cytometric analyses comparing the expression of mesenchymal surface marker proteins CD73, CD90 and CD105 and the negative markers such as CD34 and CD45 in the iMSCs and UC‐MSCs at the passage 9 (P9). There are significant changes observed between iMSCs (C) and UC‐MSCs (D) in the expression of MSC‐specific proteins. Isotype control antibodies were used as negative control

In order to compare the MSC‐specific gene and protein expression pattern in iMSCs and UC‐MSCs at P7, we performed qRT‐PCR and flow cytometry analyses. The qRT‐PCR data show that there is no significant difference in MSC‐specific genes except CD90. The level of CD90 gene expression is significantly higher in UC‐MSCs when compared to iMSCs. Moreover, among the expression of MSC‐negative markers there is a markedly increased expression of CD45 in UC‐MSCs (Figure 5A). When we compared these cells at a higher passage (P18), we have observed that the level of gene expressions of CD73 and CD105 was significantly higher in iMSCs when compared to UC‐MSCs (Figure 5B). The MSCs from two sources were analysed by flow cytometry to compare the phenotypic expression of MSC‐specific surface proteins CD73, CD90 and CD105, and the negative markers CD34 and CD45. Our data demonstrated that the positive MSC‐specific markers CD73, CD90 and CD105 were highly expressed in both cell types studied. Moreover, the iMSCs and UC‐MSCs were shown a low expression of MSC‐negative markers CD34 and CD45 (Figure 5C,D). These results suggested that both cells are equally good in the expression of MSC‐specific mRNAs and proteins.

### 3.7 No significant difference in proliferation, growth and colony formation in iMSCs and UC‐MSCs

**FIGURE 6:**
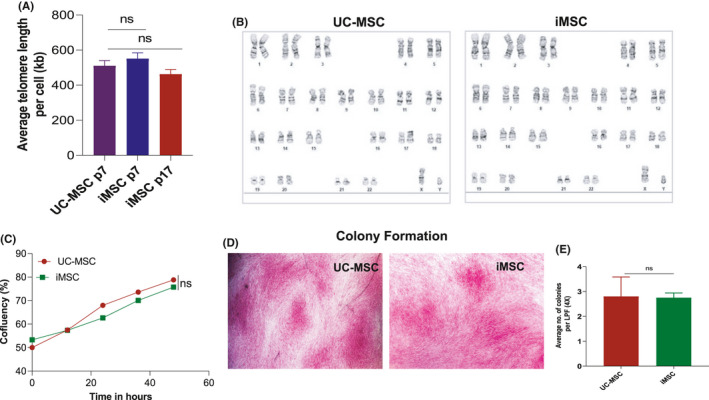
Phenotypic and functional comparison of iMSCs with UC‐MSCs. (A) Absolute human telomere length quantification was performed (as specified in Methods) in iMSCs and UC‐MSCs at the early (P7) and late passage (P17) cells. (B) G‐band karyotyping analysis showed that both UC‐MSC and UE‐iMSC had a normal karyotype (46,XY) and no clonal abnormalities were detected at the band level of resolution 375–400. (C) The degree of confluency was measured in iMSCs and UC‐MSCs under the same specific condition at P8. The cell growth was examined by live imaging microscope for 48 h. Based on the number of adherent cells on a 2‐dimensional cell culture plate, area within the field of view was recorded. No significant changes were observed in cell growth between iMSCs and UC‐MSCs. (D) Colony‐forming unit (CFU) assay was performed with iMSCs and UC‐MSC at the P7. The colonies were identified by crystal violet staining, and the colonies were stained positive as shown in microscopic images. (E) The stained colonies were counted in at least 20 frames taken at 4× magnification with EVOS microscope per dish. There were no significant changes in the stained colonies between iMSCs and UC‐MSCs; ns, not significant

Telomere length at the end of chromosomes ultimately defines the proliferative capacity of a cell. The status of telomeres is an important parameter for MSC quality, and the telomere lengths can be used in specific selection of the MSCs and can be used as a quality control measure to select the desired MSCs from a culture.^24^ Hence, we have measured the distribution of telomere lengths in the iMSCs and UC‐MSC cell population. To analyse the absolute telomere length in different MSCs, the iMSCs’ early (P7) and late passages (P17) and UC‐MSCs (P7) cells were collected and their DNAs were isolated. The isolated DNA was analysed using absolute human telomere length quantification qPCR assay as described in the Methods. Our results showed the telomere length was similar between iMSCs and UC‐MSCs (Figure 6A). Importantly, no significant difference in telomere length was observed between the early (P7) and late passages (P17) of iMSCs (Figure 6A). The telomere length suggested that both MSCs have long telomere which can enhance their proliferative potential.

In order to analyse the chromosomal abnormalities, which may happen during the long culture of cells, we performed G‐banded karyotyping analysis using iMSCs and UC‐MSCs at their passage 15. The analysis showed a normal karyotype (46,XY) with no clonal abnormalities were detected in both UC‐MSCs and iMSCs (Figure 6B). Live imaging of the iMSC is instrumental in revealing the cell growth and quantification of the confluency. For that iMSCs, UC‐MSC was cultured under same specific condition at the passage 8. Measuring the number of adherent cells on a 2‐dimensional cell culture plate area within the field of view by live imaging allows for quantification of the degree of confluency. The measurement of the confluency of iMSCs and UC‐MSC for 48 h revealed that there was no significant difference in the cell growth pattern between these cells (Figure 6C).

The colony‐forming unit (CFU) assay was used to study the cellular survival and growth assessment of the differences in reproductive viability. The CFU assay is now widely used as an efficient method to quantify stromal progenitors.^25^, ^26^ This ability of colony formation is one of the key characteristics of MSC to determine their proliferation potential. CFU assay was performed with iMSCs and UC‐MSCs at the passage 7. The MSCs were seeded into a 30‐mm dish at a density of 6.25 × 10^4^ cells with the MesenCult medium for 14 days as described earlier. Small‐ to medium‐sized colonies were visible after 7 days of culture. The cultures were maintained for 14 days, and the individual colonies were distinguished by crystal violet staining (Figure 6D). The stained colonies were counted in at least 20 frames taken per dish at 4× magnification with EVOS microscope. We observed that there was no significant difference between iMSCs and UC‐MSCs in the number of colonies formed (Figure 6E).

### 3.8 Anti‐inflammatory properties of iMSCs and UC‐MSCs

**FIGURE 7:**
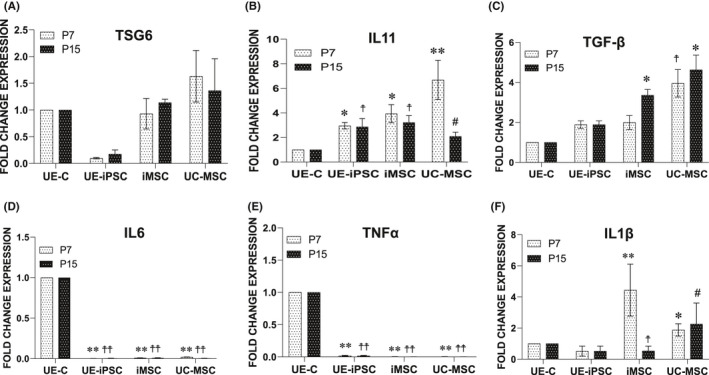
Comparison of the anti‐inflammatory potentials of UE‐iMSCs with UC‐MSC. (A–F) The mRNA expression of anti‐inflammatory (IL‐11, TGF‐β and TSG‐6) and pro‐inflammatory (IL‐6, IL1β and TNF‐α) genes was examined using qRT‐PCR in iMSCs and UC‐MSCs at the early passage (P7) and the late passage (P15). (A) The mRNA expression of anti‐inflammatory gene TSG‐6 is higher in UC‐MSCs when compared to iMSCs, and the difference is not statistically significant. (B) The mRNA expression of anti‐inflammatory gene IL‐11 is higher in UC‐MSCs, iMSCs and iPSCs when compared to UE‐C than iMSCs. In iPSCs and iMSCs, the IL‐11 gene expression is consistent in the early and late passages, whereas in UC‐MSCs, the IL‐11 gene expression is significantly reduced at the late passage. *p < 0.05 vs. UE‐C at P7; ^†^ *p *< 0.05 vs. UE‐C at P15; **p < 0.01 vs. UE‐C at P7; ^#^<0.05 iMSCs vs. UC‐MSCs at P15. (C) The mRNA expression of anti‐inflammatory gene TGF‐β is significantly higher in the late iMSCs and both early and late passage UC‐MSCs when compared to UE‐C. ^†^ *p *< 0.05 vs. UE‐C, UE‐iPSCs and iMSCs at P7; *p < 0.05 vs. UE‐C and UE‐iPSCs at P15. (D and E) The mRNA expression of pro‐inflammatory gene IL‐6 and TNF‐α were significantly decreased in the late the early and late passages of iPSCs, iMSCs and UC‐MSCs when compared to UE‐C. **p < 0.01 vs. UE‐C at P7; ^††^ *p *< 0.01 vs. UE‐C at P15. (F) The mRNA expression of pro‐inflammatory gene IL1β is significantly increased in the early passage of iMSCs and UC‐MSCs when compared to iPSCs and UE‐C. The IL1β gene is significantly reduced in iMSC late passage when compared to the early passage as well as UC‐MSCs. *p < 0.05 vs. UE‐C and UE‐iPSCs at P7; **p < 0.01 vs. UE‐C and UE‐iPSCs at P7; ^†^ *p *< 0.01 vs. UC‐MSCs at P15; ^#^ *p *< 0.05 vs. UE‐C, UE‐iPSCs and iMSCs at P15

The anti‐inflammatory cytokines are the immunoregulatory molecules that regulate the pro‐inflammatory cytokines response. MSC that possess anti‐inflammatory effects has been shown to have therapeutic advantages in preclinical studies. In order to study the anti‐inflammatory potentials of MSCs, the constitutive expression of anti‐ and pro‐inflammatory marker proteins was studied using iMSC and UC‐MSC at the early (P7) and the late (P15) passages. Gene expressions of major anti‐inflammatory cytokines such as IL‐11, TGF‐β and TSG‐6, and pro‐inflammatory cytokines such as IL‐6, IL1β and TNF‐α were studied in the above two cell populations. Our results have shown that the gene expressions of anti‐inflammatory cytokines IL‐11, TGF‐β and TSG‐6 were higher in both the MSCs compared with the UE‐C cells (Figure 7A–C). The mRNA expression of pro‐inflammatory gene IL‐6 and TNF‐α was significantly decreased in the early and late passages of iPSCs, iMSCs and UC‐MSCs when compared to UE‐C (Figure 7D,E). The mRNA expression of pro‐inflammatory gene IL1β was significantly increased in the early passage of iMSCs and UC‐MSCs when compared to iPSCs and UE‐C. The IL1β gene was significantly reduced in the late passage of iMSCs when compared to the early passage, whereas in UC‐MSCs, even at the late passage, an increased levels of IL1β gene expression were maintained (Figure 7F).

### 3.9 iMSCs and its conditioned medium had a superior cell migration capacity than the UC‐MSCs and its conditioned medium

**FIGURE 8:**
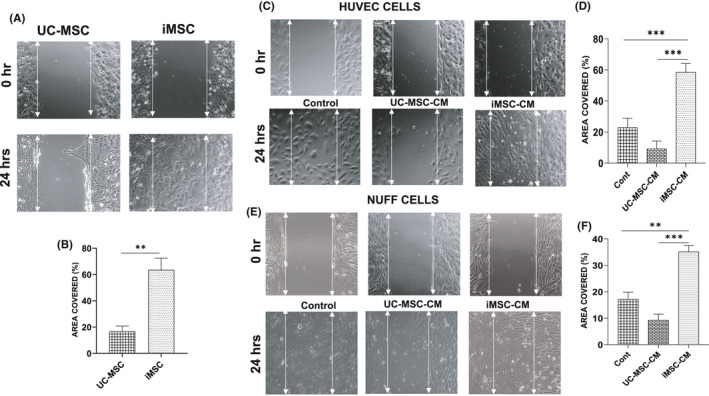
The migration and proliferative potentials of conditioned medium from iMSC and UC‐MSC using scratch assay. (A and B) Migration properties of UC‐MSC and iMSC were measured using the scratch assay (A), and the graph (B) shows the percentage of scratch area covered by the iMSCs and UC‐MSC measured using ImageJ software. (C) Scratch assay performed using HUVECs. The phase‐contrast images showed that the scratch made in HUVECs cells and cultured under condition medium closed faster than the HUVECs cultured in control medium and the condition medium from UC‐MSCs. (D) The graph showed that the percentage of area covered by the condition media from iMSCs are better than control medium or UC‐MSC–conditioned medium. (E) Scratch assay performed using NUFF. The phase‐contrast image analysis of area covered by NUFF using different media. (F) Similar to the HUVECs, the graph showed that the percentage of area covered by the condition media from iMSCs are better than control medium or UC‐MSC–conditioned medium. The scratch area was measured using ImageJ software

Scratch assay is an in vitro method to assess the cell migration under specific culture condition. iMSC showed a significantly higher migratory capacity when compared to UC‐MSC (Figure 8A,B). Similarly, the migratory effect of the conditioned medium from iMSCs showed significantly higher migration potential in both HUVECs (Figure 8C,D) and NUFF cells (Figure 8E,F) when compared to the conditioned medium from UC‐MSCs. In addition, the Transwell migration assay showed that iMSC had significantly higher migratory capacity than UC‐MSC (Figure S4). These results clearly suggested that the iMSCs and its conditioned medium possessed a superior migration capacity in covering the scratch area as well as transmigrating ability through the porous membrane than the UC‐MSCs and its conditioned medium.

## 4 DISCUSSION

MSCs are increasingly used for regenerative therapies under multiple disease conditions. Treatment with MSC is promising for various degenerative diseases mainly because of their multilineage differentiation, modulation of local niches through immunomodulation and paracrine secretion properties.^27^, ^28^, ^29^ In general, MSCs have a low expression of class I MHC and no expression of class II MHC along with a low level of co‐stimulatory molecules such as CD80, CD40 and CD86. These characteristics of MSCs provide inhibition of cytotoxic T‐cell invasion.^30^ However, currently, MSCs face some limitations which include fewer number of cells with low expandability potentials, allogenic immunogenicity and an invasive procedure for obtaining autologous MSCs which hinder their application potential. Moreover, studies have shown that iPSC‐derived MSCs have engrafted well and improved kidney injury,^31^ limb ischaemia^32^ and allergic rhinitis.^33^ Thus, our aim is to utilize the autologous UE‐iMSCs for treating patients with various diseases without any immunological reactions. Since teratoma formation is more common with the iPSCs,^15^ the use of iMSCs is a better source of cells for therapy. These iPSC‐derived MSCs are highly proliferative and non‐immunogenic.^32^ But in most of these cases, invasive surgical procedures are required for obtaining adult MSCs or somatic cells, which are used for reprogramming into iPSCs. In the current study, we have developed an easy, non‐invasive and inexpensive method for obtaining autologous epithelial cells from human urine, and further employed a safe non‐viral method of reprogramming them into iPSCs^16^ followed by their differentiation into iMSCs. Previously, UE cells have been isolated from human newborn urine^34^ and from adults.^35^ However, their growth characteristics were very limited. Several years later urine‐derived stem‐like cells (UDSC) have been identified^36^; nevertheless, the source of UDSCs and the expression levels of their surface markers are controversial.^37^ In this study, we have demonstrated that the UE‐derived iMSCs have good proliferative, migratory and multilineage differentiation capacities without losing the characteristics of MSCs, even at late passages.

Our comparative study results based on the expression of MSC surface markers, functional characteristics such as growth curve analysis, colony formation assay and maintaining the telomere length even at late passage suggest that the UE‐derived autologous iMSCs were equally good as the UC‐MSCs. However, iMSCs maintained their MSC surface markers even at the later passage (P18), during which the UC‐MSCs started to lose its positive MSC markers such as CD73, CD90 and CD105, and began to gain hematopoietic markers such as CD34 and CD45. These results indicate that the qualities of UC‐MSCs are good at the early passage, but expanding and using them for cell therapy, especially at later passage may pose the risk of losing its functional characteristics. On the other hand, iMSCs maintain their characteristic features even at later passages.

Mostly, UC‐MSCs are used for allogenic therapy, and there are some possibilities for the development of an immune reaction.^38^, ^39^ In a randomized control study, systemic immune reactions such as increased plasma pentraxin‐3, IL8 and TLR4 were observed in cerebral palsy patients treated with umbilical cord blood cells.^39^ However, iPSC‐derived MSCs have been known for their immunomodulatory characteristics through downregulation of M1 macrophages and upregulation of M2 macrophages during cardiopulmonary resuscitation.^40^ TSG‐6 is shown to have various tissue‐protective and anti‐inflammatory properties and mediates many of the immunomodulatory and beneficial activities of mesenchymal stem/stromal cells.^41^, ^42^, ^43^ IL‐11 was shown to induce MSCs towards proliferation, migration and attenuation of apoptosis.^44^ Overexpression of TGF‐β1 in human synovium‐derived MSCs enhanced their proliferation and chondrogenic differentiation potentials.^45^ Based on the above studies, we have analysed the expression of TSG‐6, IL‐11 and TGFβ1 in iMSC and UC‐MSC at the early and late passages. The expressions of TSG‐6, IL‐11 and TGFβ were enhanced in iMSC even at later passage. Predominantly, IL‐6 has been shown as a pro‐inflammatory molecule,^46^ and we have consistently observed that IL‐6 mRNA expression is low in iMSC and UC‐MSC. Currently, it is not clear why IL‐1B expression is higher in iMSCs at the early passage (p7). However, a recent report indicated that priming of human MSCs with interleukin‐1 induces them towards an anti‐inflammatory and pro‐trophic phenotype in vitro.^47^ These results indicate that the generated iMSCs have enhanced anti‐inflammatory, immunomodulatory and proliferative potentials even at the late passages. Furthermore, the migration potentials of the iMSC, UC‐MSC and their conditioned medium were evaluated through scratch assay. The migration capacity of the iMSCs and its conditioned medium was significantly higher when compared to the UC‐MSCs and its conditioned medium. These data in agreement with the previous findings with UC‐MSCs^48^ clearly suggested that the wound‐healing property is better using iMSCs than UC‐MSCs.

In this study, we have generated UE‐derived autologous iMSCs, which are superior in maintaining the characteristics of MSC, at both the early and late passages, whereas the MSC characteristics have been declined in UC‐MSCs. Moreover, storage of umbilical cord blood cells is very expensive, and the long‐term storage of them may result in the loss of its functional characteristics. This expensive long‐term storage can be avoided through the application of autologous iMSC therapy. Moreover, at the basal condition, UE cells express a low level of NANOG, and this may result in an easier reprogramming of UE cells when compared to the adult fibroblasts.

In conclusion, we have developed non‐invasive, safe, non‐immunogenic, autologous iMSCs, which are highly proliferating and maintaining its MSC characteristics without any chromosomal abnormalities even at the later passage. Our comparative study of iMSCs with UC‐MSCs has shown that the iMSCs were equally good or even better than UC‐MSCs for the same functions. Furthermore, iMSCs can provide an unlimited supply of cells which will be a suitable source of cells for developing clinical‐grade autologous cells for therapy.

## CONFLICTS OF INTEREST

The authors declare no potential conflict of interest relevant to this article.

## AUTHOR CONTRIBUTIONS

**Sheeja Rajasingh:** Conceptualization (equal); Data curation (equal); Formal analysis (equal); Methodology (equal); Software (equal); Writing‐original draft (equal). **Vinoth Sigamani:** Data curation (equal); Formal analysis (equal); Investigation (equal); Methodology (equal). **Vijay Selvam:** Data curation (equal); Formal analysis (equal); Investigation (equal); Methodology (equal). **Narasimman Gurusamy:** Data curation (equal); Formal analysis (equal); Investigation (equal); Methodology (equal); Writing‐original draft (equal); Writing‐review & editing (equal). **Shivaani Kirankumar:** Data curation (equal); Formal analysis (equal); Investigation (equal); Methodology (equal). **Jayavardini Vasanthan:** Data curation (equal); Formal analysis (equal); Investigation (equal); Methodology (equal). **Johnson Rajasingh:** Conceptualization (lead); Data curation (supporting); Funding acquisition (lead); Investigation (equal); Methodology (equal); Project administration (lead); Resources (lead); Supervision (lead); Validation (lead); Visualization (equal); Writing‐original draft (lead); Writing‐review & editing (equal).

## Supporting information

Fig S1‐4

Table S1‐3

Supplementary Material

## Data Availability

The data supporting the findings of this study are available from the corresponding author upon reasonable request.
