# Supplementary figures and images for: Comparative analysis of human induced pluripotent stem cell‐derived mesenchymal stem cells and umbilical cord mesenchymal stem cells
Source: J Cell Mol Med. 2021 Aug 13;25(18):8904–19. doi: 10.1111/jcmm.16851 (PMC8435459; doi:10.1111/jcmm.16851)

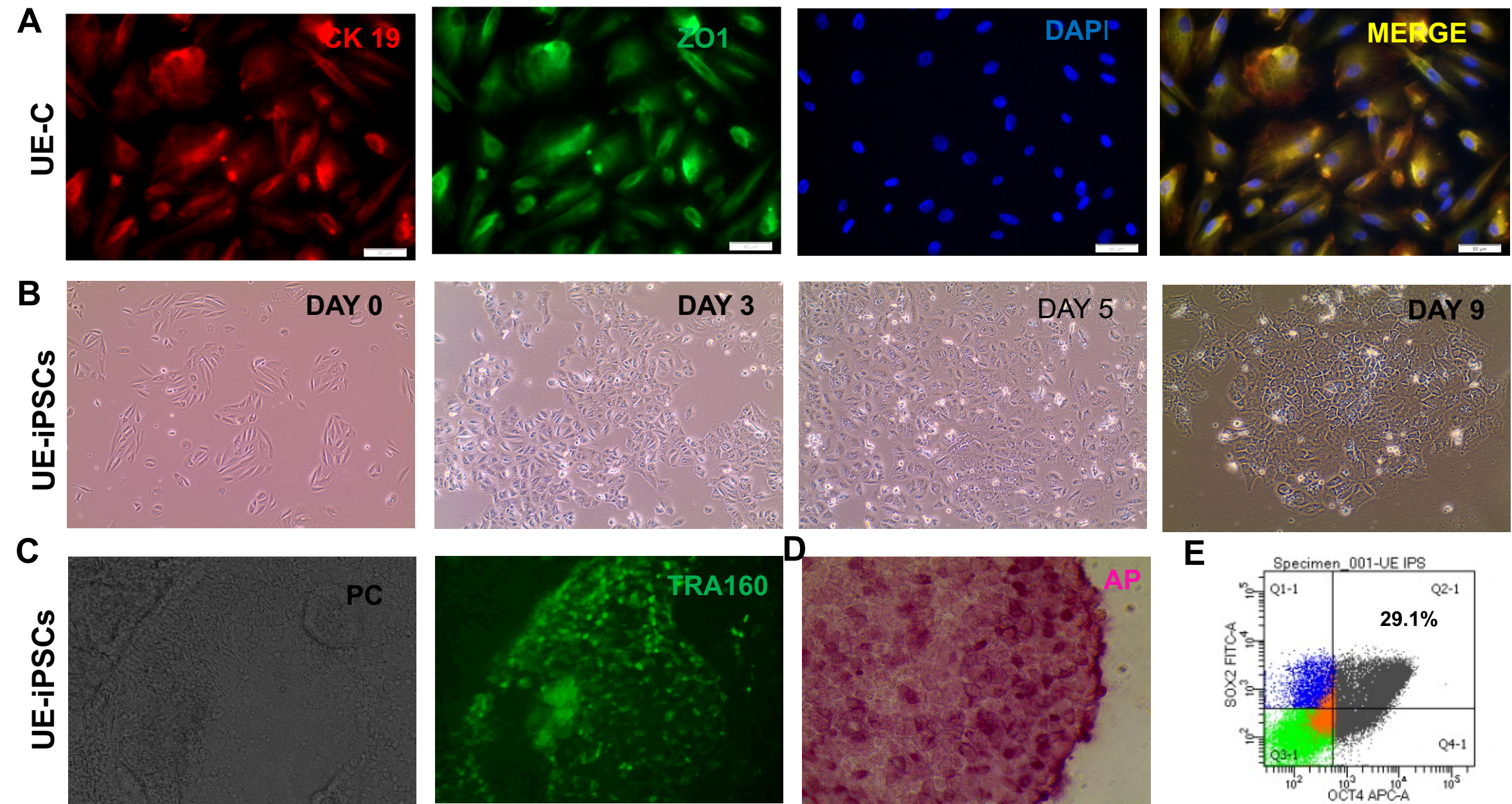

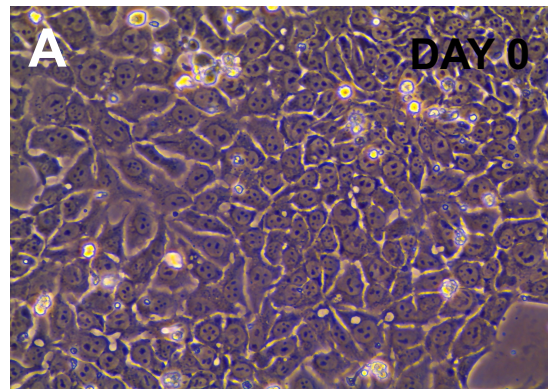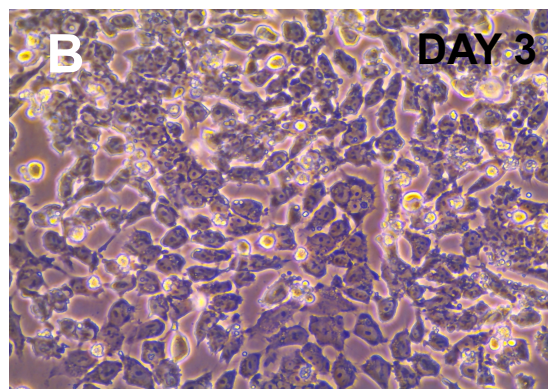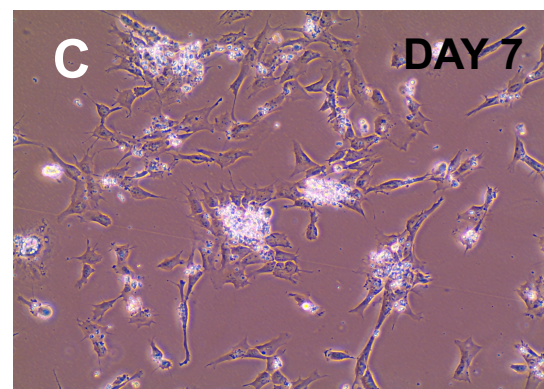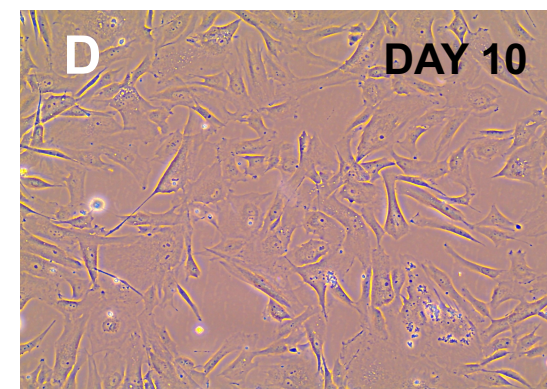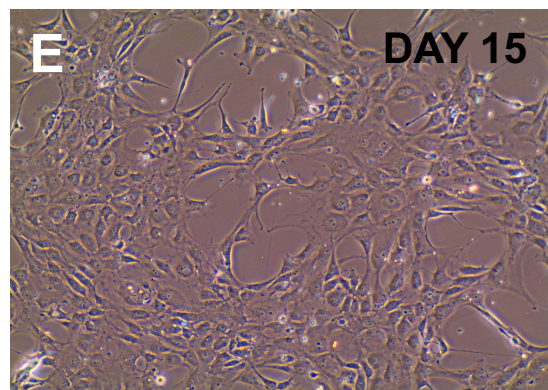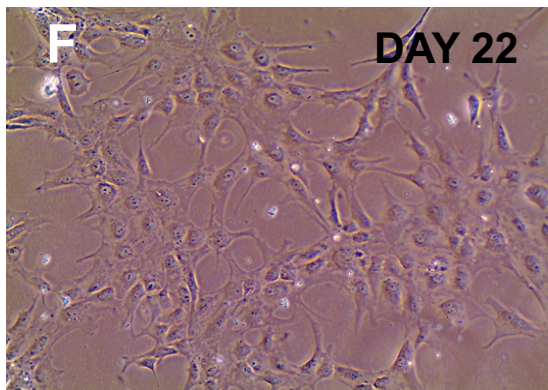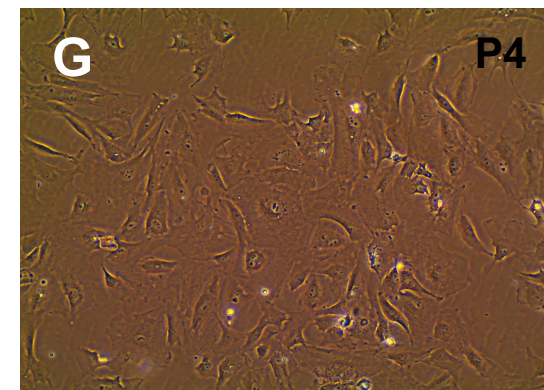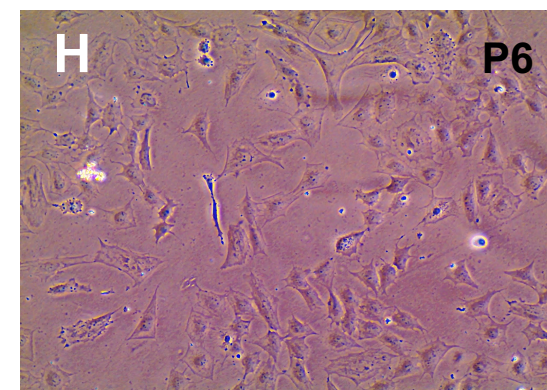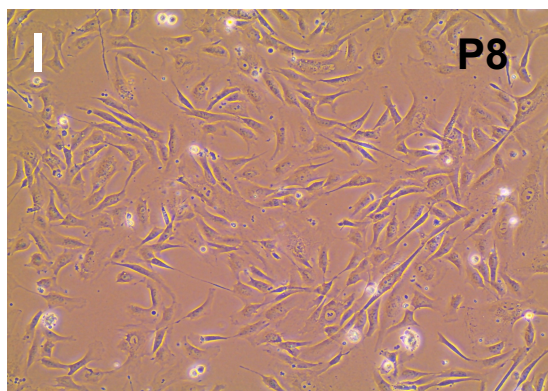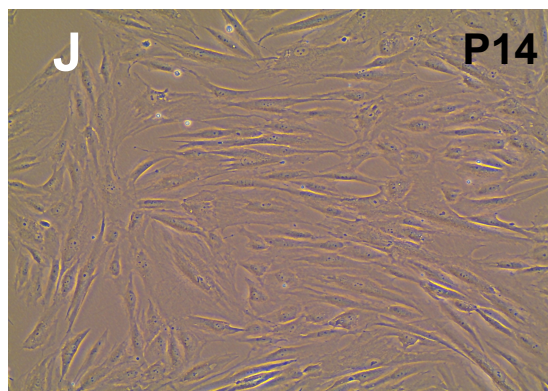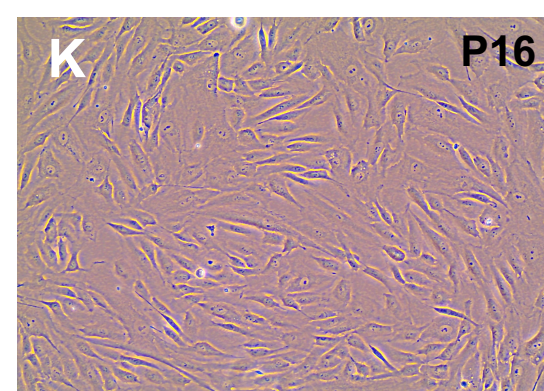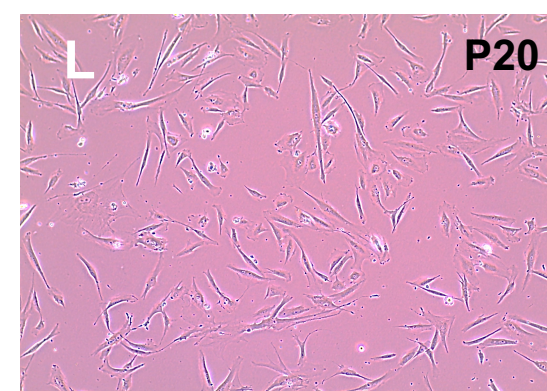

iMSCs

iOST

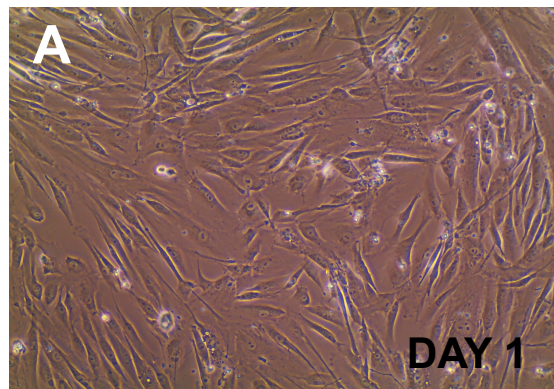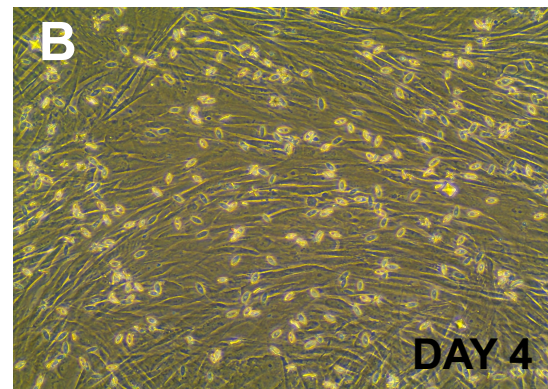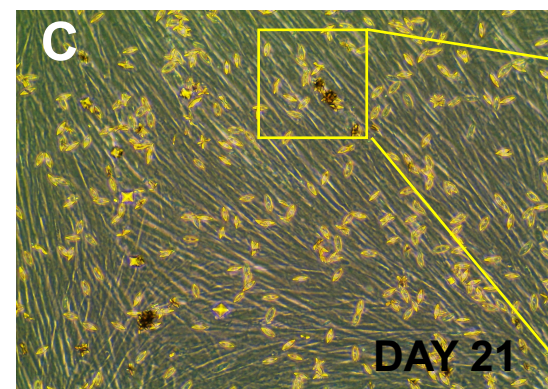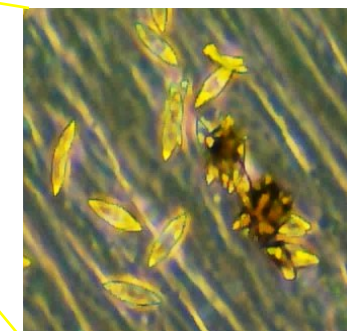

iCHON

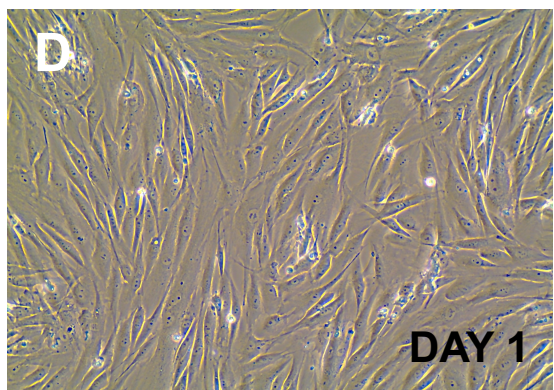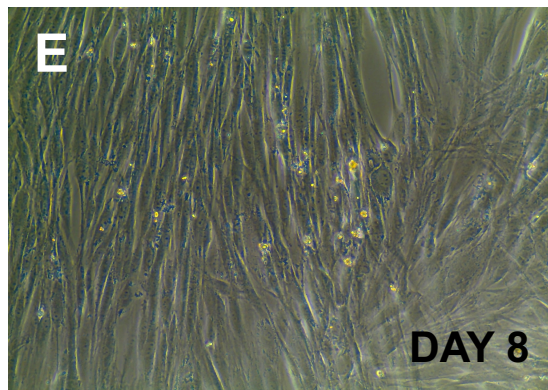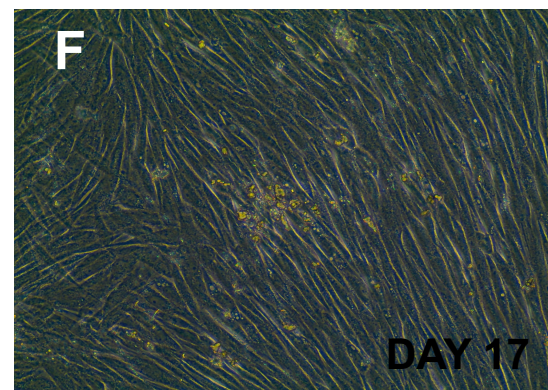

iADIPO

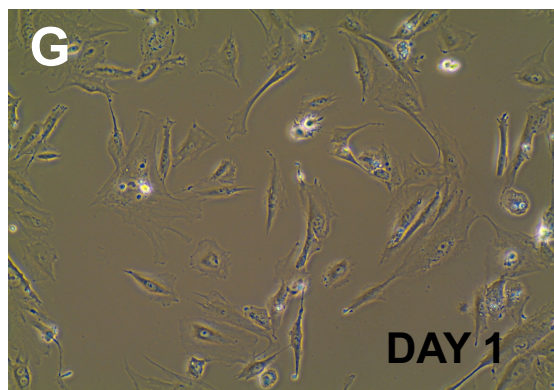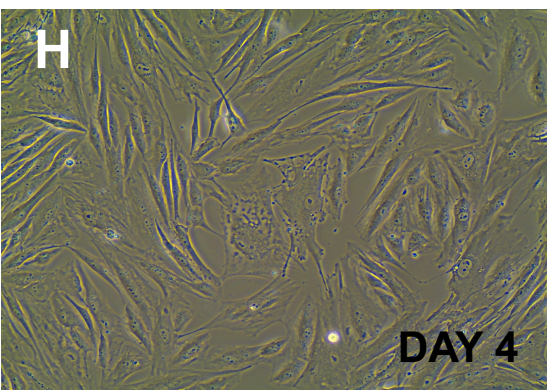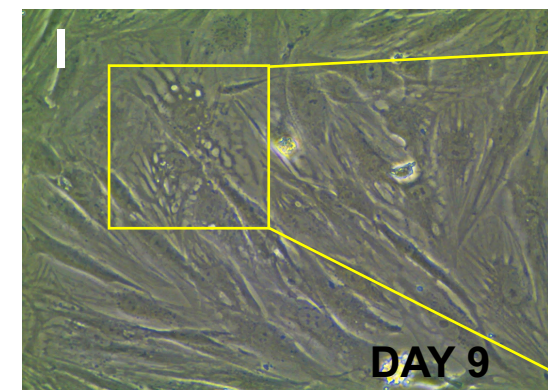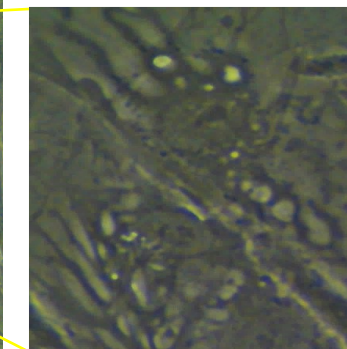

Supplementary Figure S3

**A**

**iMSC**

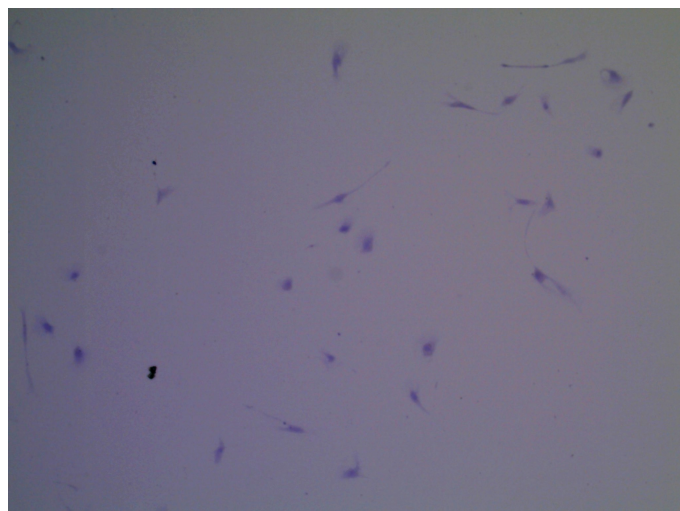

**UC-MSC**

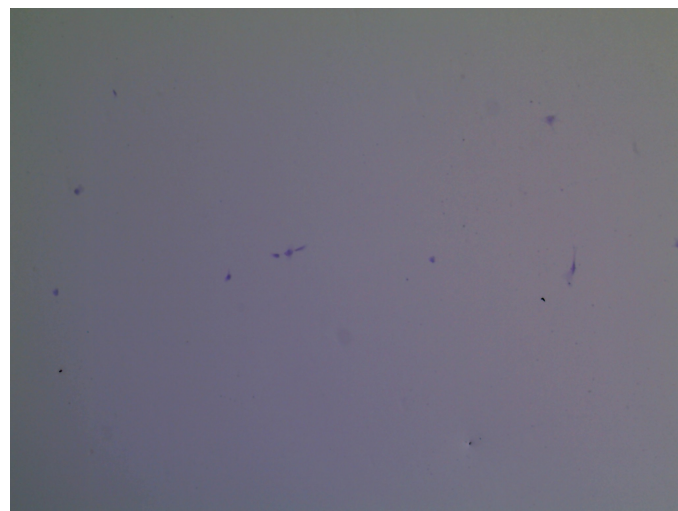

**B**

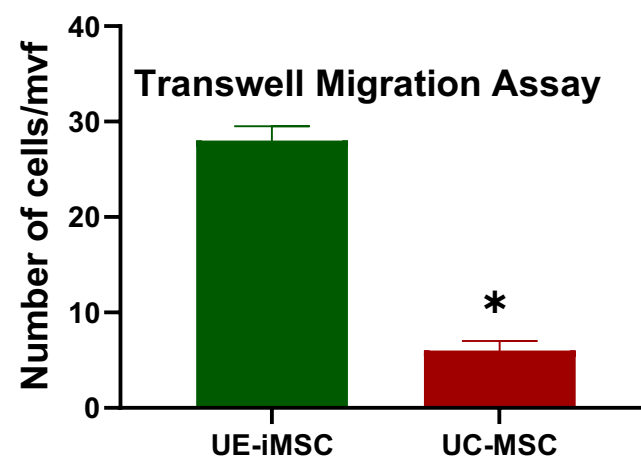

Supplement: Supplementary file 1 — Fig S1‐4 [file JCMM-25-8904-s002.pdf]
