# Supplementary material for: Comparative analysis of human induced pluripotent stem cell‐derived mesenchymal stem cells and umbilical cord mesenchymal stem cells
Source: J Cell Mol Med. 2021 Aug 13;25(18):8904–19. doi: 10.1111/jcmm.16851 (PMC8435459; doi:10.1111/jcmm.16851)
Supplement: Supplementary file 2 — Table S1‐3 [file JCMM-25-8904-s001.pdf]

Online Supplementary Tables

Supplementary Table 1. Details of antibodies used in this study

Gene	Cat.#	Company	Used
SSEA4	ASA-0150	Applied Stem Cell	IF
OCT3/4	SC-5279	Santa cruz	IF,WB
SOX2	SC-365964	Santa cruz	IF,WB
NANOG	SC-293121	Santa cruz	IF,WB
OCT3/4	130-105-080	Miltenyi Biotec	FACS
SOX2	130-104-993	Miltenyi Biotec	FACS
NANOG	130-109-764	Miltenyi Biotec	FACS
H3	9715	cell signalling	WB
VECAD	SC-8399	Santa cruz	IF
AFP	SC-8399	Santa cruz	WB
NESTIN	MAB1259	R&D System	WB
ACTIN	SC-1616	Santa cruz	WB
CD73	NBP2-48480SS	Novus Biologicals	IF
CD90	NBP2-66763	Novus Biologicals	IF,WB
CD105	NBAF1097	Novus Biologicals	IF
CD73	SC-32299	Santa cruz	WB
CD73-APC	130-112-061	Miltenyi Biotec	FACS
CD90-PE	130-117-537	Miltenyi Biotec	FACS
CD105-FITC	130-112-327	Miltenyi Biotec	FACS
CD34-FITC	11-034182	eBioscience	FACS
CD45-PE	561087	BD Bioscience	FACS
IgG_APC	405308	Biolegends	FACS
IgG_FITC	405305	Biolegends	FACS
IgG_PE	405307	Biolegends	FACS
CK19	ab52625	abcam	IF
ZO1	SC-8147	Santa cruz	IF

Supplementary Table 2. SYBR Green Primer list		
**Gene**	**Forward**	**Reverse**
**18S rRNA**	5` -CTACCACATCCAAGGAAGCA- 3`	5` -TTTTTCGTCACTACCTCCCCG- 3`
**OCT3/4**	5` -ACCCCTGGTGCCGTGAA- 3`	5` -GGCTGAATACCTTCCCAAATA- 3`
**SOX2**	5` -CAGCGCATGGACAGTTAC- 3`	5` -GGAGTGGGAGGAAGAGGT- 3`
**NANOG**	5` -AAAGGCAAACAACCCACT- 3`	5` -GCTATTCTTCGGCCAGTT- 3`
**VECAD**	5` -CCTGATGCGGCTAGGCATA- 3`	5` -GGAAGAACTGGCCCTTGTCA- 3`
**CD31**	5` -TGTATTTCAAGACCTCTGTGCACTT- 3`	5` -TTAGCCTGAGGAATTGCTGTGTT- 3`
**CD73**	5` -CAGTACCAGGGCACTATCTGG- 3`	5` -AGTGGCCCCTTTGCTTTAAT- 3`
**CD90**	5` -ATGAACCTGGCCATCAGCA- 3`	5` -GTGTGCTCAGGCACCCC- 3`
**CD105**	5` -CCACTAGCCAGGTCTCGAAG- 3`	5` -GATGCAGGAAGACACTGCTG- 3`
**CD34**	5` -TCTTGGGCATCACTGGCTATT - 3`	5` -GCCCAGCCTTTCTCCTGTG- 3`
**CD45**	5` -TGGTAAAAGCTCTACGCAAAGCT- 3`	5` -AGGGTAGGTGCTGGCAATGA- 3`
**OSTEOCALCIN**	5` -ATGAGAGCCCTCACACTCCTC- 3`	5` -GCCGTAGAAGCGCCGATAGGC- 3`
**COL2**	5` -GGCAATAGCAGGTTCACGTACA- 3`	5` -CGATAACAGTCTTGCCCCACTT- 3`
**ADIPONECTIN**	5` -AAGGAGATCCAGGTCTTATTGG- 3`	5` -ACCTTCAGCCCCGGGTAC- 3`
**TSG6**	5` -TCACCTACGCAGAAGCTAAGGC- 3`	5` -TCCAACTCTGCCCTTAGCCATC- 3`
**IL11**	5` -GGACCACAACCTGGATTCCCTG- 3`	5` -AGTAGGTCCGCTCGCAGCCTT- 3`
**TGF-β**	5` -GCAGCACGTGGAGCTGTA- 3`	5` -CAGCCGGTTGCTGAGGTA- 3`
**IL6**	5` -AGACAGCCACTCACCTCTTCAG- 3`	5` -TTCTGCCAGTGCCTCTTTGCTG- 3`
**IL1β**	5` -TGAGCTCGCCAGTGAAATGA- 3`	5` -AGATTCGTAGCTGGATGCCG- 3`
**TNFα**	5` -CAGCCTCTTCTCCTTCCTGAT- 3`	5` -GCCAGAGGGCTGATTAGAGA- 3`

Table 2

Supplementary Table: 3<br>MSC taqman Primer list	
**Gene**	**Primer_ID**
**18s**	Hs03928985_g1
**APOA1**	Hs01040607_m1
**AFP**	Hs01040607_m1
**MAP2**	Hs00258900_m1
**OLIG2**	Hs00300164_s1
