# Supplementary material for: Comparative analysis of human induced pluripotent stem cell‐derived mesenchymal stem cells and umbilical cord mesenchymal stem cells
Source: J Cell Mol Med. 2021 Aug 13;25(18):8904–19. doi: 10.1111/jcmm.16851 (PMC8435459; doi:10.1111/jcmm.16851)
Supplement: Supplementary file 3 — Supplementary Material [file JCMM-25-8904-s003.docx]

**Online Supplementary materials**

**Supplemental Figure S1. Non-viral reprogramming of human urinary epithelial cells derived induced pluripotent cells. A**, Human urinary epithelial cells (UE) cells, identified through the expression of marker proteins K19 and ZO1 were cultured in a 25 ml flask containing DMEM medium. **B**, When these cells become 80% confluent, they were sub-cultured into a 6-well plate coated with iMatrix with NutriStem medium. Then the cells were reprogrammed for 10 days using StemRNA Reprogramming kit as described in Methods. The appearance of iPSCs cells on different days such as day-0, day-3, day-7, and day-9 during the reprogramming of UECs. From day 9, we have observed several iPSC granulated colonies resembled human embryonic stem cell colonies. **C**, These UE-derived iPSC colonies generated were identified through phase contrast microscope (PC) and manually picked by live staining with TRA160 and further these iPSCs were confirmed by alkaline phosphatase staining (AP).

**Supplemental Figure S2. Generation of UE-iPSC-derived induced MSCs (iMSCs). A-F**, For the differentiation of UE-iPSCs into iMSCs, UE-iPSC were cultured in NutriStem XF/FF culture medium until they 70% confluency, then the medium was removed and fresh mesenchymal induction medium (STEMdiff-ACF Mesenchymal Induction Medium) was added to the plates. Media was changed for every day for 21 days.  Phase contrast microscopic images of cells during different days of differentiation (A-F). **G-L,** The iMSCs were maintained and subcultured in Mesencult ACF Medium. Phase contrast microscopic images showing iMSCs during various passages (G-L).

**Supplemental Figure S3. A-C, iMSCs trilineage sequential morphological changes. A-C,** The sequential morphological changes from iMSC to iOST were observed under phase contrast microscope. **D-F,** The sequential morphological changes from iMSC to iCHON were observed under a phase contrast microscope. **G-I,** The sequential morphological changes from iMSC to iADIPO were observed under a phase contrast microscope.

**Supplemental Figure S4. A**, Microscopic images showing the migration capacity of iMSC and UC-MSC measured using the transwell migration assay, where the number of cells migrated to the surface of the culture plate was stained with 0.5% crystal violet. **B**, The number of transwell-migrated iMSCs and UC-MSCs were counted using an inverted phase-contrast microscope under 20X visual field (mvf). *p<0.05
